# Readiness to the privatization of the health system in Saudi Arabia: Translation and factor analysis of Readiness to Organizational change (ROC) scale

**DOI:** 10.1371/journal.pone.0322406

**Published:** 2025-06-02

**Authors:** Ahmed Ali Alasiri, Qi Zhang

**Affiliations:** 1 Public Health Department, College of Health Sciences, Saudi Electronic University, Riyadh, Saudi Arabia; 2 Health Science College, Health Science Research, Old Dominion University, Norfolk, Virginia, United States of America; King Saud University Medical City, SAUDI ARABIA

## Abstract

**Background:**

The Kingdom of Saudi Arabia (KSA) began a major transformation as a part of version 2030 for its health system. These reform initiatives aim to privatize health services and improve efficiency. Despite this meaningful change, there is a knowledge gap in how healthcare providers prepare for or perceive this shift. Specifically, there has been a lack of validation of Arabic instruments that gauge the individual readiness to this change. This study bridges the gap by localizing and validating the Readiness to Organizational Change scale (ROC) to provide empirical evidence and to understand adaptability for the KSA context.

**Method:**

The study aimed to test and validate a tool that can be used to assess the ability of individuals to embrace the change initiatives among healthcare providers. A cross-sectional study was conducted in a public healthcare setting that included allied healthcare providers and physicians and excluded the administration staff. Ethical approval was obtained, and the questionnaire was distributed among participants electronically.

**Result:**

There were 296 participants who completed the survey, with (78.0%) being male and they were between 25–35 years old, while the females represented (22%). The majority had less than 10 years’ experience and were married (88.2%). The face and content validity showed an excellent result for Arabic context S-CVI/AVE = 0.97. The inter-item correlation revealed a significant value (p < 0.001), which confirmed convergent validity, and Factor analysis assessed the adequacy of sampling (KMO = 0.77). The Exploratory Factor Analysis (EFA) extracted seven factors describing 74.6% of the total Variance, with an adequate value of commonalities for most items.

**Conclusion:**

The Arabic version of ROC is a valid and reliable scale for assessing readiness among healthcare providers in the context of KSA. This translated version can provide insightful information and facilitate the transition of the health system in KSA and achieve the healthcare vision of 2030.

## Introduction

The healthcare industry is a dynamic and challenging environment for professionals delivering high-quality health services. Hospitals are constantly exposure to new information, diverse care styles, governmental structures, rules and regulations, and the introduction of technologies [1]. Health organizations or hospitals also encounter other transformative events, often involving behavioral and social changes for health staff [2]. However, change initiatives in the healthcare industry have shown limited success in implementation [3]. Understanding the level of readiness among employees is a vital part of healthcare setting transformation [4]. There are several definitions and scales used to gauge readiness for change [5]. However, readiness to change is a multidimensional construct that affects beliefs among team members regarding their ability to change, the leader’s commitment to change, the benefits expected from the change for employees, and the appropriateness of the change for the organization [6]. Readiness to organizational change is one of the most prevalent attitude studies in the literature on organizational change, and considerable effort is being made to develop instruments to evaluate readiness dimensions [7,8]. Several existing measures focus on the organizational change phenomenon, such as ORIC [4]. This report focuses on the individual level because the individual is the first layer to accept or reject change activities, and only the individual of organization can forecast the success of the initiatives for change through their act [9]. The supra level represents the collective attitude toward change, which essentially includes individuals. While the healthcare system is undergoing a privatization shift in Saudi Arabia and the Ministry of Health initiated a transformation plan for its health system to ensure sustainability for its population [10], there is no valid scale in the Saudi context that measures the individual readiness to change. Also, the validation is context dependent [7]. Therefore, there is a need to bridge the gap in measurement that addresses readiness levels among healthcare teams. Holt et al. developed a readiness to change scale that addresses change at the individual level. The scale is grounded in a framework, undergoes psychometric testing, and is applicable in a healthcare setting [11]. The scale consists of four main constructs with 25 questions: appropriateness (10 items) – whether the healthcare team considers the change appropriate for their hospital or not; change efficacy (6 items) – the ability of team members to handle the change; management support (6 items) – the support provided by hospital leaders for the healthcare team to implement the proposed change; and personal valence (3 items) – the personal benefits of the proposed change. Furthermore, the ROC scale has been tested and applied in different countries and settings [12,13], and the scale showed a superior rate among other scales for individual readiness due to its rigorous psychometric test and applicability in various settings[6,7,11]. Since the healthcare system in Saudi Arabia is undergoing a significant health transformation [10], measuring the readiness level using a reliable and valid scale will support the success of the change and facilitate the process of achieving the strategic goals of the Ministry of Health in the kingdom. Additionally, the translated version might serve as a first step for each department in a health organization, whether big or small, planning to update its policy or introduce new services or information. Also, it will illustrate the factors that hinder change efforts by health authorities through an understating of the four main constructs of the Holt formwork. This report aims to examine the Arabic version of the readiness to change scale developed by Holt, and the validity and reliability of the version will be assessed in a hospital setting. Arabic language is one of the most spoken languages globally in more than 18 countries and around 400 million people [14].

## Methods

### Participants and procedure

The study was conducted in the general hospital in the Asser region of Saudi Arabia. The study included physicians and allied health providers. Healthcare providers were involved if they had an active practicing license aged 18 years or older, and there were no restrictions on gender and nationality; however, the report excluded health administration staff since this main goal was to assess the frontline of health providers. The study utilized a cross-sectional study. The directorate of health affairs in the Asser region granted ethical approval to IRB log No: REC-2-7-2023. The instrument was piloted to 10 healthcare providers to ensure the sustainability of each item. The pre-test used a self-administrated method, and the researcher included a personal phone number in case there were misleading items or questions that participants misunderstood. The invitation letter included a study aim with a summary of the study. Also, it involved information about voluntary participation in the study, with the right to withdraw at any time. The participants have the option either to “agree” or” disagree “to participate, so when they agree that will be considered as authorization to contribute to this project. The survey didn’t intend to include any medical intervention, so a waiver for formal consent from the IRB was requested. The members of the organization are identified by the employee list provided by the manager of the hospitals. The questionnaires were managed electronically through Google Forms, and the link was sent via social applications. The link was available for six months (01/December/2023–31/Ma/ 2024), and a reminder message was sent every two weeks to encourage health employees to participate.

### Measures

The readiness to organizational change (ROC) instrument contains 25 items and four main dimensions developed by Hot et al. to gauge the readiness at the individual level within organization [11]. The scale was slightly modified to be a 5-Likert scale instead of the 7-Likert scale, in which (1= = strongly disagree and 5 = strongly agree). The 5- Likert scale was used for several purposes: to increase the number of responses and encourage participants to complete the survey. It is easier to understand by responding, and it eliminates missing some items [15].To make analysis easier, we classified and sorted the elements based on their domain.

### Translation

**Fig 1:**
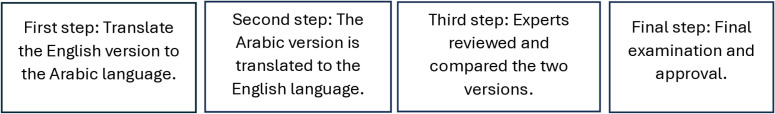
Visual summary of the four-step translation process used to develop the Arabic version of the scale. The bilingual experts agreed on the final version, which was used for the pilot study. Original and translated version (S1 Table).

The original version of the ROC is in the English language [6], so the version was translated into standard Arabic language using the Squires et al. framework [16]. The process of translation includes four main steps and is illustrated in Fig 1. First, the qualifying bilingual was translated the English version to the Arabic version. Secondly, The Arabic version was translated back to the English language. The Third step was to give the two versions of English and Arabic to the Billanuge experts to examine the accuracy. The experts checked them independently, and they discussed the findings if they any discrepancies between the two versions by comparing them. In the final step, the version was assessed, and it was found to be valid, and it has identical meaning to the English version.

### Statistical analysis

Three types of validity tests were included to investigate the validation of Arabic version of the ROC. First, four healthcare providers (two physicians and two nurses) met to review the translation version of the ROC scale and made their decision about the most suitable translation version. Second, Content validity was obtained by four processional panels who are experts in validating behavior scales in the Arabic context. They assessed if the translated version fit the Arabic culture, and they evaluated if the format of each item was conceptually similar to the English version by following two steps [17]. First, a four-point Likert scale was utilized to test the cultural relevance (which 1 = not relevant and 4 = highly relevant). Secondly, the binary yes and no answers assessed whether each item of the Arabic version was equivalent to the original version. The second validation is content validity indexed (CVI) score were computed for culture relevance and equivalency of the scale. Third, the inter-item correlation was utilized to test the construct validity [18], so the Pearson correlation for each item will be assessed, and the value between 0.3 to 0.49 is considered a moderate correlation, and the value of > 0.5 indicates a strong value [19]. Furthermore, Exploratory factor analysis (EFA) was employed in this study to test the dataset and provide additional insight [13]. Therefore, the initial two steps of the factor analysis were conducted: a) assessment of the suitability of the data and b) factor extraction. The suitability of the data for the (EFA), Kaiser-Meyer-Olkin value (KMO), and Bartlett’s Sphericity Test were applied [20]. Factor extraction with the Principal Component Analysis (PCA) utilized to obtain the minimum factors needed to represent the data set effectively, it followed by orthogonal factor rotation as an extraction method [21]. Lastly, to examine the reliability of each of the subscales, Cronbach alpha (α) was conducted to assist the internal consistency. The Cronbach alpha (α) value ranges from 0 to 1, and since there is no global accepted range for reliability, the study will take the alpha value of 0.7 and above as accepted value [22]. IBM SPSS statistics software version 27.0. was used for analysis (Armonk, New York, USA) [23].

## Results

### Characteristics of participants

The sociodemographic characteristics of the study participants (N = 296) are summarized in Table 1. The sample was predominantly male, with 78.0% (n = 231) identifying as such, while females constituted 22.0% (n = 65) of the participants. Age distribution showed that nearly half of the respondents were between 25 and 35 years old (44.3%, n = 131). This was followed by those aged 36–45 years (35.5%, n = 105), 46–55 years (15.9%, n = 47), and more than 56 years (4.4%, n = 13). Notably, there were no participants under the age of 25. Marital status revealed that a significant majority were married (88.2%, n = 261), with smaller proportions being single (8.4%, n = 25) or divorced (3.4%, n = 10).

**Table 1:** Sociodemographic variables (N = 296).

		Count	%
Gender Age	Male	231	78.0%
	Female	65	22.0%
	25–35 years	131	44.3%
	36–45 years	105	35.5%
	46–55 years	47	15.9%
	More than 56 years	13	4.4%
Marital Status	Single	25	8.4%
	Married	261	88.2%
	Divorced	10	3.4%
Professional Speciality (Occupation)	Physician	67	22.6%
	Allied Healthcare Provider	229	77.4%
Nationality	Saudi	277	93.6%
	Non-Saudi	19	6.4%
Level of Education	Diploma	114	38.5%
	Bachelor’s degree	103	34.8%
	Master’s degree	12	4.1%
	Doctorate degree	67	22.6%
Years of Experience	Less than 5 years	36	12.2%
	5-10 years	96	32.4%
	11-15 years	68	23.0%
	16-20 years	58	19.6%
	21-25 years	7	2.4%
	More than 25 years	31	10.5%

In terms of professional specialty, 22.6% (n = 67) of the participants were physicians, while a substantial 77.4% (n = 229) were allied healthcare providers. The nationality of the participants was predominantly Saudi, with 93.6% (n = 277) identifying as such and 6.4% (n = 19) being non-Saudi. Regarding the level of education, the sample included individuals with diverse educational backgrounds: 38.5% (n = 114) had a diploma, 34.8% (n = 103) held a bachelor’s degree, 4.1% (n = 12) had a master’s degree, and 22.6% (n = 67) possessed a doctorate. The distribution of years of experience among participants showed that 12.2% (n = 36) had less than 5 years of experience, 32.4% (n = 96) had 5–10 years, 23.0% (n = 68) had 11–15 years, 19.6% (n = 58) had 16–20 years, 2.4% (n = 7) had 21–25 years, and 10.5% (n = 31) had more than 25 years of experience.

### Questionnaires validation

We conducted descriptive analysis for each item of the scale for the total number of participants, 291. The mean value ranged from 3.25 to 3.79, and the range of the standard deviation was from 0.77 to 1. 73. Table 2 showed the item description and commonalitles for all items were above the threshold of the 0.50, indication a good proportion of the Variance among the items.

**Table 2:** Descriptive of ROC items and communities.

Variables		Mean score (SD)	Communities
Item	**Appropriateness Construct**		
Q1		3.64 ± 1.73	0.803
Q2		3.67 ± 1.20	0.894
Q3		3.53 ± 1.18	0.884
Q4		3.42 ± 1.08	0.765
Q5		3.56 ± 1.06	0.834
Q6		3.29 ± 0.99	0.624
Q7		3.54 ± 1.03	0.712
Q8		3.52 ± 1.15	0.734
Q9		3.27 ± 1.50	0.778
Q10		3.25 ± 0.97	0.776
Item	**Management Support Construct**		
Q1		3.56 ± 0.77	0.803
Q2		3.29 ± 0.93	0.602
Q3		3.33 ± 0.99	0.799
Q4		3.60 ± 1.10	0.795
Q5		3.37 ± 0.95	0.743
Q6		3.56 ± 1.10	0.822
Item	**Change Efficacy Construct**		
Q1		3.45 ± 0.91	0.633
Q2		3.36 ± 1.03	0.657
Q3		3.69 ± 0.94	0.577
Q4		3.30 ± 1.12	0.671
Q5		3.46 ± 1.04	0.762
Q6		3.79 ± 0.91	0.821
Item	**Personal Valance Construct**		
Q1		3.26 ± 0.93	0.621
Q2		3.25 ± 1.03	0.813
Q3		3.25 ± 0.85	0.659

### Face and content validity

The result of the meeting for face validity between physicians and nurses revealed some minor changes to improve the readability of the overall of scale. The recommendation of the team was to produce a final version. The content validity Score showed an adequate level of cultural relevance, and the overall S-CVI/AVE of the translated version was excellent 0.97. Therefore, all items of the final translated versions were utilized to assess readiness among healthcare providers. (S2 Table).

### Convergent validity

The inter-item correlation for the subscale was significant at (p < 0.001). Likewise, the Pearson correlation value for most items showed a strong value, exceeding 0.5. Therefore, the four constructs of the ROC are valid (S3 Table).

### Exploratory factor analysis

To test the adequacy of the data, the output of the Kaiser-Meyer-Olkin (KMO = 0.77) and Bartlett’s Test of Sphericity (X^2^ = 539.266, p < 0.000), as shown in Table 3 which confirmed that the data are suitable for EFA.

**Table 3:** KMO and Bartlett’s Test.

Kaiser-Meyer-Olkin Measure of Sampling Adequacy.		.775
Bartlett’s Test of Sphericity	Approx. Chi-Square	5395.266
	df	300
	Sig.	<.001

### Factor extraction

Kaiser’s criterion is employed to determine initial unrotated components that need to be extracted. Table 4 shows the total Variance explained by the extracted factors. Prior to the extraction, twenty-five items are revealed from the data set, and after extraction, there are seven factors. They explained a cumulative variance of 74.61. The Principal Component Analysis method is the extraction technique used in this study with the varimax rotation. The first component showed 29.8% of the total Variance and an eigenvalue of 7.7. The second component has explained 12.02 of the variances with 3.4 eigenvalues. The component third to seven have shown lower than 10% of total Variance for each of them. Specifically, they explained 8.45%, 5.19%, 3.67%, 3.00%, and 2.56% of the Variance, respectively, with eigenvalues ranging from 2.43 to 1.03.

**Table 4:** The eigenvalues and Total Variance Explained.

Total Variance Explained						
Factor	Initial Eigenvalues			Extraction Sums of Squared Loadings		
	Total	% of Variance	Cumulative %	Total	% of Variance	Cumulative %
1	7.718	30.870	30.870	7.468	29.873	29.873
2	3.404	13.615	44.486	3.005	12.020	41.893
3	2.430	9.719	54.205	2.113	8.453	50.346
4	1.698	6.791	60.995	1.299	5.198	55.544
5	1.264	5.056	66.051	.919	3.675	59.219
6	1.101	4.405	70.456	.751	3.004	62.223
7	1.039	4.155	74.611	.641	2.564	64.787
8	.838	3.352	77.963			
9	.737	2.948	80.910			
10	.690	2.761	83.671			
11	.623	2.490	86.162			
12	.535	2.139	88.300			
13	.492	1.970	90.270			
14	.421	1.683	91.953			
15	.354	1.417	93.371			
16	.317	1.268	94.639			
17	.268	1.071	95.710			
18	.250	1.000	96.710			
19	.206	.826	97.535			
20	.166	.665	98.200			
21	.134	.536	98.735			
22	.120	.479	99.214			
23	.090	.359	99.574			
24	.067	.268	99.842			
25	.040	.158	100.000			

Extraction Method: Principal Component Factoring.

### Reliability

The overall internal consistency of the scale was 0.85. Table 5 summarizes the reliability of the four constructs of the ROC scale, indicating the number of items in each scale and their respective Cronbach’s alpha coefficients.

**Table 5:** Reliability of Scales.

Scale	Number of items	Cronbach’s Alpha
Appropriateness	10	0.781
Personal Valence	3	0.752
Management Support	6	0.773
Change Efficacy	6	0.698

## Discussion

While the attitude of the individual to change is a major reason for the failure of change initiatives [24,25] however, to the best of our knowledge, there are no studies in the KSA that tackle the individual readiness for the adoption of privatization and healthcare transformation. The present paper attempts to bridge the knowledge gap and provide a translated instrument to examine the ability of individuals to change. Individuals in an organization are a main component and will provide valuable preliminary data for the policymakers. The main aim of the study was to psychometric test the Arabic version of the ROC scale among healthcare providers in KSA. The study evaluated several types of validity, including face and content validity, and to establish convergent validity, the (EFA) was done. Additionally, the reliability of the scale was evaluated using Cronbach’s alpha. Based on our results, the Arabic version has similar outcomes to the original English version [11]. VI technical utilized in this study, however, as best to our knowledge, is one of the strengths points of our study since the CVI has not been employed in any psychometric research testing the properties of ROC. The CVI confirmed that the translated version has equivalent and semantically meaning to the original English version. The internal consistency value was high for appropriateness α = 0.78, Management support α = 0.77, and personal value α = 0.75 however only change efficacy dimensions was slightly low α = 0.69. The original version of ROC showed a higher α value for appropriateness α = 0.94, management support α = .087, change efficacy α = 0.82, and only personal value was = α.066. which is similar to the study that evaluated the readiness to the new indicator of quality and safety among health care providers in the primary health center, the value of Cronbach alpha was over the threshold for all dimensions except the personal value was α = 0.66 [26]. Similarly to the study that tested the readiness among clinical providers and non-clinical in Australia to the hospital redevelopment, they included two dimensions, appropriateness, and change efficacy; the value was α = 0.85 and 0.75, respectively [12]. The validation of the Arabic version has significant implications for the healthcare sector in the KSA since the health system is under transformation to enhance the delivery of health, so this scale can help the administration member in the health cluster or at the hospital level either big or small to identify area of the resistance and to enable tailored change management strategy. While the overall reliability is over the threshold of 0.7, further research on refining the change efficacy construct could improve its consistency value. Also, this study was conducted in only one health center, so further validation research would enhance the generalizability in the Saudi health context. Additionally, based on the fact that we used the standard Arabic translation, the translated version should be understood, and it can be utilized in any Arabic country.

## Conclusion

The study demonstrates strong validity and reliability for the Arabic version of the ROC, making it a valuable tool for evaluating readiness among individuals in healthcare settings. Consequently, policymakers could find it practical and helpful to test the individual ability to change and tailor change strategies. It will provide insightful information for the leader that supports the overall goal of Vision 2030 for the health system and contribute to the long-term success of the change initiatives. Similarly, the Arabic version can aid the significant transformation of the organization by identifying approaches to help minimize the disruption and provide quality health services to patients.

## Supporting information

S1 Table The original English and Arabic versions of the ROC questionnaire. (PDF)

S2 Table Content Validity Index CVI. (PDF)

S3 Table Inter-Item correlation of translated version of ROC. (PDF)
