# Supplementary material for: Readiness to the privatization of the health system in Saudi Arabia: Translation and factor analysis of Readiness to Organizational change (ROC) scale
Source: PLoS One. 2025 Jun 2;20(5):e0322406. doi: 10.1371/journal.pone.0322406 (PMC12129205; doi:10.1371/journal.pone.0322406)
Supplement: S1 Table — (PDF) [file pone.0322406.s001.pdf]

Q No.	Question		Level of measurement	Coding for responses				
رقم السؤال	السؤال		مستوى القياس	ترميز الاجابات				
1-	Gender:	الجنس:	Nominal اعتباري	1-	Male	☐	ذكر	-1
				2-	Female	☐	أنثى	-2
2-	Your Age is:	العمر:	Ordinal ترتيبي	1-	Less than 25 years	☐	أقل من ٢٥ سنة	-1
				2-	25 – 35 years	☐	بين ٢٥-٣٥ سنة	-2
				3-	36 – 45 years	☐	بين ٣٦-٤٥ سنة	-3
				4-	46 – 55 years	☐	بين ٤٦-٥٥ سنة	-4
				5-	More than 56 years	☐	أكبر من ٥٦ سنة	-5
3-	Marital Status:	الحالة الاجتماعية:	Nominal اعتباري	1-	Single	☐	أعزب	-1
				2-	Married	☐	متزوج	-2
				3-	Divorced	☐	مطلق	-3
4-	Profession:	المهنة :	Nominal اعتباري	1-	Physician	☐	طبيب	-1
				2-	Allied Healthcare Provider	☐	مقدم رعاية صحية بالتخصصات الطبية المساعدة	-2
	If Allied Healthcare Provider, please identify your professional background: إذا كنت من مقدمي الرعاية الصحية بالتخصصات الطبية المساعدة، فيرجى تحديد مهنتك:			1-	Nurse	☐	ممرض	-1
				2-	Pharmacist	☐	صيدلي	-2
				3-	Audiologist	☐	فني سمعيات	-3
				4-	Dental Hygienist	☐	أخصائي صحة الفم والأسنان .	-4
				5-	Cardiovascular Technologist	☐	أخصائي تقنيات القلب والأوعية الدموية.	-5
				6-	Physiotherapist	☐	فني علاج طبيعي	-6
				7-	Other	☐	أخرى	-7
5-	Nationality:	الجنسية :	Nominal اعتباري	1-	Saudi	☐	سعودي	-1
				2-	Non -Saudi	☐	غير سعودي	-2
6-	Your level of Education is:	مستوى التعليم:	Ordinal ترتيبي	1-	Diploma	☐	دبلوم	-1
				2-	Bachelor’s degree	☐	بكالوريوس	-2
				3-	Master’s Degree	☐	ماجستير	-3
				4-	Doctorate degree	☐	دكتوراه	-4
7-	Your Years of Experience:	عدد سنوات الخبرة :	Ordinal ترتيبي	1-	Less than 5 years	☐	أقل من 5 سنوات	-1
				2-	5 – 10 years	☐	بين 5-10 سنوات	-2
				3-	11 – 15 years	☐	بين 11- 15 سنة	-3
				4-	16 – 20 years	☐	بين 16 - 20 سنة	-4
				5-	21 – 25 years	☐	بين 21 - 25 سنة	-5
				6-	More than 25 years	☐	أكبر من 25 سنة	-6

Q No.	Question	Level of measurement	Coding for responses				
رقم السؤال	السؤال	مستوى القياس	ترميز الاجابات				
**Readiness towards Health Care Transformation: Appropriateness مدى الاستعداد لتحويل الرعاية الصحية: الملاءمة**							
1-	It does not make much sense for us to initiate this change. ليس من المنطقي بالنسبة لنا البدء في هذا التغيير.	Ordinal ترتيبي	1-	Strongly disagree	☐	أعارض بشدة	-1
			2-	Disagree	☐	أعارض	-2
			3-	Neither agree or disagree	☐	لا أوافق ولا أعارض	-3
			4-	Agree	☐	أوافق	-4
			5-	Strongly agree	☐	أوافق بشدة	-5
2-	I think that the organization will benefit from this change. أعتقد ان المرفق/المستشفى سوف يستفيد من هذا التغير.	Ordinal ترتيبي	1-	Strongly disagree	☐	أعارض بشدة	-1
			2-	Disagree	☐	أعارض	-2
			3-	Neither agree or disagree	☐	لا أوافق ولا أعارض	-3
			4-	Agree	☐	أوافق	-4
			5-	Strongly agree	☐	أوافق بشدة	-5

Copyright © [2014] [Ahmed Ali Alasiri]. All rights reserved.

3-	This change makes my job easier.<br>هذا التغير سوف يجعل عملي أكثر سهولة.	Ordinal ترتيبي	1-	Strongly disagree	☐	أعارض بشدة	-1
			2-	Disagree	☐	أعارض	-2
			3-	Neither agree or disagree	☐	لا أوافق ولا أعارض	-3
			4-	Agree	☐	أوافق	-4
			5-	Strongly agree	☐	أوافق بشدة	-5
4-	This change will improve our organization's overall efficiency.<br>هذا التغيير سيعزز كفاءة مؤسستنا بشكل عام.	Ordinal ترتيبي	1-	Strongly disagree	☐	أعارض بشدة	-1
			2-	Disagree	☐	أعارض	-2
			3-	Neither agree or disagree	☐	لا أوافق ولا أعارض	-3
			4-	Agree	☐	أوافق	-4
			5-	Strongly agree	☐	أوافق بشدة	-5
5-	There are legitimate reasons for us to make this change.<br>لدينا أسباب مشروعة تجعلنا نجري هذا التغيير.	Ordinal ترتيبي	1-	Strongly disagree	☐	أعارض بشدة	-1
			2-	Disagree	☐	أعارض	-2
			3-	Neither agree or disagree	☐	لا أوافق ولا أعارض	-3
			4-	Agree	☐	أوافق	-4
			5-	Strongly agree	☐	أوافق بشدة	-5
6-	When this change is implemented, I don't believe there is anything for me to gain.<br>عندما يتم تنفيذ هذا التغيير، لا أعتقد أن هناك أي مكاسب بالنسبة لي.	Ordinal. ترتيبي.	1-	Strongly disagree	☐	أعارض بشدة	-1
			2-	Disagree	☐	أعارض	-2
			3-	Neither agree or disagree	☐	لا أوافق ولا أعارض	-3
			4-	Agree	☐	أوافق	-4
			5-	Strongly agree	☐	أوافق بشدة	-5
7-	There are a number of rational reasons for this change to be made.<br>هناك عدد من الأسباب المنطقية لتنفيذ هذا التغيير.	Ordinal. ترتيبي.	1-	Strongly disagree	☐	أعارض بشدة	-1
			2-	Disagree	☐	أعارض	-2
			3-	Neither agree or disagree	☐	لا أوافق ولا أعارض	-3
			4-	Agree	☐	أوافق	-4
			5-	Strongly agree	☐	أوافق بشدة	-5
8-	In the long run, I feel it will be worthwhile for me if the organization adopts this change.<br>على المدى الطويل، أشعر انه سيكون من المجدي بالنسبة لي تبني المؤسسة لهذا التغيير	Ordinal. ترتيبي.	1-	Strongly disagree	☐	أعارض بشدة	-1
			2-	Disagree	☐	أعارض	-2
			3-	Neither agree or disagree	☐	لا أوافق ولا أعارض	-3
			4-	Agree	☐	أوافق	-4
			5-	Strongly agree	☐	أوافق بشدة	-5
9-	The time we are spending on this change should be spent on something else<br>الوقت الذي نقضيه في هذا التغير يجب ان نقضيه في عمل شيء آخر.	Ordinal. ترتيبي.	1-	Strongly disagree	☐	أعارض بشدة	-1
			2-	Disagree	☐	أعارض	-2
			3-	Neither agree or disagree	☐	لا أوافق ولا أعارض	-3
			4-	Agree	☐	أوافق	-4
			5-	Strongly agree	☐	أوافق بشدة	-5
10-	This change matches the priorities of our organization.<br>يتوافق هذا التغيير مع أولويات المؤسسة.	Ordinal. ترتيبي.	1-	Strongly disagree	☐	أعارض بشدة	-1
			2-	Disagree	☐	أعارض	-2
			3-	Neither agree or disagree	☐	لا أوافق ولا أعارض	-3
			4-	Agree	☐	أوافق	-4
			5-	Strongly agree	☐	أوافق بشدة	-5
**Readiness towards Health Care Transformation: Management support**<br>**الاستعداد لتحويل الرعاية الصحية: دعم الإدارة**							
1 -	Our senior leaders have encouraged all of us to embrace this change.<br>القادة قاموا بتشجيع الجميع لتبني هذا التغيير.	Ordinal. ترتيبي.	1-	Strongly disagree	☐	أعارض بشدة	-1
			2-	Disagree	☐	أعارض	-2
			3-	Neither agree or disagree	☐	لا أوافق ولا أعارض	-3
			4-	Agree	☐	أوافق	-4
			5-	Strongly agree	☐	أوافق بشدة	-5

Copyright © [2014] [Ahmed Ali Alasiri]. All rights reserved.

2-	Our organization’s top decision-makers have put all their support behind this change effort.<br>لقد وضع كبار صناع القرار في مؤسستنا كل دعمهم وراء الجهود لهذا التغيير.	Ordinal.	ترتيبي.	1-	Strongly disagree	☐	أعارض بشدة	1-
				2-	Disagree	☐	أعارض	2-
				3-	Neither agree or disagree	☐	لا أوافق ولا أعارض	3-
				4-	Agree	☐	أوافق	4-
				5-	Strongly agree	☐	أوافق بشدة	5-
3-	Every senior manager has stressed the importance of this change.<br>أكد كل المدراء الكبار على أهمية هذا التغيير.	Ordinal.	ترتيبي.	1-	Strongly disagree	☐	أعارض بشدة	1-
				2-	Disagree	☐	أعارض	2-
				3-	Neither agree or disagree	☐	لا أوافق ولا أعارض	3-
				4-	Agree	☐	أوافق	4-
				5-	Strongly agree	☐	أوافق بشدة	5-
4-	I think we are spending a lot of time on this change when the senior managers don’t even want it implemented.<br>أعتقد أننا نقضي الكثير من الوقت في هذا التغيير في حين أن كبار المديرين لا يريدون حتى تنفيذه.	Ordinal.	ترتيبي.	1-	Strongly disagree	☐	أعارض بشدة	1-
				2-	Disagree	☐	أعارض	2-
				3-	Neither agree or disagree	☐	لا أوافق ولا أعارض	3-
				4-	Agree	☐	أوافق	4-
				5-	Strongly agree	☐	أوافق بشدة	5-
5-	This organization’s most senior leader is committed to this change.<br>القائد العام لهذه المؤسسة ملتزم بهذا التغيير.	Ordinal.	ترتيبي.	1-	Strongly disagree	☐	أعارض بشدة	1-
				2-	Disagree	☐	أعارض	2-
				3-	Neither agree or disagree	☐	لا أوافق ولا أعارض	3-
				4-	Agree	☐	أوافق	4-
				5-	Strongly agree	☐	أوافق بشدة	5-
6-	Management has sent a clear signal this organization is going to change.<br>أرسلت الإدارة إشارة واضحة بأن هذه المؤسسة ستتغير.	Ordinal.	ترتيبي.	1-	Strongly disagree	☐	أعارض بشدة	1-
				2-	Disagree	☐	أعارض	2-
				3-	Neither agree or disagree	☐	لا أوافق ولا أعارض	3-
				4-	Agree	☐	أوافق	4-
				5-	Strongly agree	☐	أوافق بشدة	5-
**Readiness towards Health Care Transformation: Change Efficacy**<br>**الاستعداد لتحول الرعاية الصحية: فعالية التغيير**								
1 -	I do not anticipate any problems adjusting to the work I will have when this change is adopted.<br>لا أتوقع وجود أي مشاكل في التكيف مع العمل الذي سأقوم به عندما يتم اعتماد هذا التغيير.	Ordinal.	ترتيبي.	1-	Strongly disagree	☐	أعارض بشدة	1-
				2-	Disagree	☐	أعارض	2-
				3-	Neither agree or disagree	☐	لا أوافق ولا أعارض	3-
				4-	Agree	☐	أوافق	4-
				5-	Strongly agree	☐	أوافق بشدة	5-
2-	When we implement this change, I feel I can handle it with ease.<br>عندما ننفذ هذا التغيير، أشعر أنني أستطيع التعامل معه بسهولة.	Ordinal.	ترتيبي.	1-	Strongly disagree	☐	أعارض بشدة	1-
				2-	Disagree	☐	أعارض	2-
				3-	Neither agree or disagree	☐	لا أوافق ولا أعارض	3-
				4-	Agree	☐	أوافق	4-
				5-	Strongly agree	☐	أوافق بشدة	5-
3-	When I set my mind to it, I can learn everything that will be required when this change is adopted.<br>عندما أعقد العزم، أستطيع تعلم كل ما هو مطلوب عندما يعتمد هذا التغيير.	Ordinal.	ترتيبي.	1-	Strongly disagree	☐	أعارض بشدة	1-
				2-	Disagree	☐	أعارض	2-
				3-	Neither agree or disagree	☐	لا أوافق ولا أعارض	3-
				4-	Agree	☐	أوافق	4-
				5-	Strongly agree	☐	أوافق بشدة	5-
4-	There are some tasks that will be required when we change that I don’t think I can do well.<br>هناك بعض المهام التي ستكون مطلوبة عندما نقوم بالتغيير ولا أعتقد أنني سأقوم بها بشكل جيد.	Ordinal.	ترتيبي.	1-	Strongly disagree	☐	أعارض بشدة	1-
				2-	Disagree	☐	أعارض	2-
				3-	Neither agree or disagree	☐	لا أوافق ولا أعارض	3-
				4-	Agree	☐	أوافق	4-
				5-	Strongly agree	☐	أوافق بشدة	5-

Copyright © [2014] [Ahmed Ali Alasiri]. All rights reserved.

5-	I have the skills that are needed to make this change work.<br><br>لدي المهارات اللازمة لإنجاح هذا التغيير.	Ordinal. ترتيبي.	1-	Strongly disagree	☐	أعارض بشدة	-1
			2-	Disagree	☐	أعارض	-2
			3-	Neither agree or disagree	☐	لا أوافق ولا أعارض	-3
			4-	Agree	☐	أوافق	-4
			5-	Strongly agree	☐	أوافق بشدة	-5
6 -	My past experiences make me confident that I will be able to perform successfully after this change is made.<br><br>تجربتي السابقة تجعلني واثقًا من أنني سأكون قادرًا على الأداء بنجاح بعد إجراء هذا التغيير.	Ordinal. ترتيبي.	1-	Strongly disagree	☐	أعارض بشدة	-1
			2-	Disagree	☐	أعارض	-2
			3-	Neither agree or disagree	☐	لا أوافق ولا أعارض	-3
			4-	Agree	☐	أوافق	-4
			5-	Strongly agree	☐	أوافق بشدة	-5
**Readiness towards Health Care Transformation:** ***personal Valence***<br>**الجاهزية نحو تحول الرعاية الصحية: القيمة الشخصية**							
1 -	I am worried that I will lose some of my status in the organization when this change is implemented.<br>أخشى أن أفقد بعضًا من مكانتي في المنظمة عند تنفيذ هذا التغيير	Ordinal. ترتيبي.	1-	Strongly disagree	☐	أعارض بشدة	-1
			2-	Disagree	☐	أعارض	-2
			3-	Neither agree or disagree	☐	لا أوافق ولا أعارض	-3
			4-	Agree	☐	أوافق	-4
			5-	Strongly agree	☐	أوافق بشدة	-5
2-	This change will disrupt many of the personal relationships that I have developed.<br><br>هذا التغيير سيعطل العديد من العلاقات الشخصية التي طورتها.	Ordinal. ترتيبي.	1-	Strongly disagree	☐	أعارض بشدة	-1
			2-	Disagree	☐	أعارض	-2
			3-	Neither agree or disagree	☐	لا أوافق ولا أعارض	-3
			4-	Agree	☐	أوافق	-4
			5-	Strongly agree	☐	أوافق بشدة	-5
3-	My future in this job will be limited because of this change.<br><br>سيكون مستقبلي في هذه الوظيفة محدودًا بسبب هذا التغيير.	Ordinal. ترتيبي.	1-	Strongly disagree	☐	أعارض بشدة	-1
			2-	Disagree	☐	أعارض	-2
			3-	Neither agree or disagree	☐	لا أوافق ولا أعارض	-3
			4-	Agree	☐	أوافق	-4
			5-	Strongly agree	☐	أوافق بشدة	-5

Copyright © [2014] [Ahmed Ali Alasiri]. All rights reserved.
