# Supplementary material for: Readiness to the privatization of the health system in Saudi Arabia: Translation and factor analysis of Readiness to Organizational change (ROC) scale
Source: PLoS One. 2025 Jun 2;20(5):e0322406. doi: 10.1371/journal.pone.0322406 (PMC12129205; doi:10.1371/journal.pone.0322406)
Supplement: S2 Table — (PDF) [file pone.0322406.s002.pdf]

The rating of relevance completed by 4 experts in validating healthcare scale.

Item	Constructs	A	B	C	D	No. of Agreement	i-CVI	UA
	Appropriateness	A	B	C	D	No. of Agreement	i-CVI	UA
**Q1**		1	1	1	1	4	1	1
**Q2**		1	1	1	1	4	1	1
**Q3**		1	1	1	1	4	1	1
**Q4**		1	0	1	1	3	0.75	0
**Q5**		1	1	1	1	4	1	1
**Q6**		1	1	1	1	4	1	1
**Q7**		1	1	1	1	4	1	1
**Q8**		1	1	1	1	4	1	1
**Q9**		1	1	1	0	3	0.75	0
**Q10**		1	1	1	1	4	1	1
	Management Support							
**Q11**		1	1	1	0	3	0.75	0
**Q12**		1	1	1	1	4	1	1
**Q13**		1	1	1	1	4	1	1
**Q14**		1	1	1	1	4	1	1
**Q15**		1	1	1	1	4	1	1
**Q16**		1	1	1	1	4	1	1
	Change Efficacy							
**Q17**		1	1	1	1	4	1	1
**Q18**		1	1	1	1	4	1	1
**Q19**		1	1	1	1	4	1	1
**Q20**		1	1	1	1	4	1	1
**Q21**		1	1	1	1	4	1	1
**Q22**		1	1	1	1	4	1	1
	Personal Valance							
**Q23**		1	1	1	1	4	1	1
**Q24**		1	1	1	1	4	1	1
**Q25**		1	1	1	1	4	1	1

S-CVI/AVE	0.97	
S-CVI/UA	0.88	

The S-CVI AVE, and S-CVI/UA meet satisfactory level for the Arabic scale.

Copyright © [2014] [Ahmed Ali Alasiri]. All rights reserved.
