# Supplementary material for: Readiness to the privatization of the health system in Saudi Arabia: Translation and factor analysis of Readiness to Organizational change (ROC) scale
Source: PLoS One. 2025 Jun 2;20(5):e0322406. doi: 10.1371/journal.pone.0322406 (PMC12129205; doi:10.1371/journal.pone.0322406)
Supplement: S3 Table — (PDF) [file pone.0322406.s003.pdf]

Table S3: Inter-item correlation of Arabic version of ROC scale

Appropriateness (Appt)

Item Pair	Correlation (r)	p-value
Highest		
Appro2 - Appro3	0.903	<0.001
Appro3 - Appro5	0.811	<0.001
Lowest		
Appro1 - Appro6	-0.089	<0.001
Appro2 - Appro10	-0.236	<0.001
Above 0.5		
Appro2 - Appro3	0.903	<0.001
Appro2 - Appro5	0.778	<0.001
Appro3 - Appro4	0.640	<0.001
Appro4 - Appro5	0.565	<0.001

Management Support (MS)

Item Pair	Correlation (r)	p-value
Highest		
MS4 - MS6	0.636	<0.001
MS5 - MS6	0.581	<0.001
Lowest		
MS2 - MS4	-0.066	0.222
MS2 - MS7	0.162	<0.001
Above 0.5		
MS4 - MS6	0.636	<0.001
MS5 - MS6	0.581	<0.001
MS1 - MS6	0.559	<0.001

Personal Valence (PV)

Item Pair	Correlation (r)	p-value
Highest		
PV1 - PV2	0.582	<0.001
PV2 - PV3	0.495	<0.001
Lowest		
PV1 - PV3	0.432	<0.001

Change Efficacy (CE)

Item Pair	Correlation (r)	p-value
Highest		
CE5 - CE6	0.534	<0.001
CE4 - CE6	0.461	<0.001
Lowest		
CE4 - CE5	0.046	0.443
CE1 - CE5	0.117	0.013
Above 0.5		
CE5 - CE6	0.534	<0.001

This table highlights the highest and lowest correlations, as well as those above a certain threshold (r > 0.5), for each construct.
